# The unique social sense of puerperium: Increased empathy and *Schadenfreude* in parents of newborns

**DOI:** 10.1038/s41598-020-62622-7

**Published:** 2020-04-01

**Authors:** Ana-María Gómez-Carvajal, Hernando Santamaría-García, Adolfo M. García, Mario Valderrama, Jhony Mejia, Jose Santamaría-García, Mateo Bernal, Jaime Silva, Agustín Ibáñez, Sandra Baez

**Affiliations:** 1 Universidad de los Andes, Bogotá, Colombia; 2 Neuroscience Research Group NEUROS, School of Medicine and Health Sciences, Universidad del Rosario, Bogotá, Colombia; 3 Memory and cognition Center, Intellectus. Hospital Universitario San Ignacio, Bogotá, Colombia; 4 Pontificia Universidad Javeriana, Departments of Physiology, Psychiatry and Aging Institute, Bogotá, Colombia; 5 Universidad de San Andrés, Buenos Aires, Argentina; 6 National Scientific and Technical Research Council (CONICET), Buenos Aires, Argentina; 7 Faculty of Education, National University of Cuyo (UNCuyo), Mendoza, Argentina; 8 Departamento de Lingüística y Literatura, Facultad de Humanidades, Universidad de Santiago de Chile, Santiago, Chile; 9 Department of Biomedical Engineering, Universidad de los Andes, Bogotá, Colombia; 10 Department of gynecology and obstetrics, Hospital Universitario San Ignacio, Bogotá, Colombia; 11 Universidad Autónoma del Caribe, Barranquilla, Colombia; 12 Center for Social and Cognitive Neuroscience (CSCN), School of Psychology, Universidad Adolfo Ibáñez, Santiago de Chile, Chile; 13 Australian Research Council Center of Excellence in Cognition and its Disorders, Sydney, Australia

**Keywords:** Emotion, Human behaviour

## Abstract

Pregnancy and puerperium are typified by marked biobehavioral changes. These changes, which are traceable in both mothers and fathers, play an important role in parenthood and may modulate social cognition abilities. However, the latter effects remain notably unexplored in parents of newborns (PNs). To bridge this gap, we assessed empathy and social emotions (envy and *Schadenfreude*) in 55 PNs and 60 controls (childless healthy participants without a romantic relationship or sexual intercourse in the previous 48 hours). We used facial electromyography to detect physiological signatures of social emotion processing. Results revealed higher levels of affective empathy and *Schadenfreude* in PNs, the latter pattern being accompanied by increased activity of the *corrugator suppercilii* region. These effects were not explained by potential confounding variables (educational level, executive functioning, depression, stress levels, hours of sleep). Our novel findings suggest that PNs might show social cognition changes crucial for parental bonding and newborn care.

## Introduction

In the course of adult development, the transition to parenthood defines a new stage marked by unique biobehavioral changes^1,2^. In particular, puerperium is a special and variable period in the life of all who become parents, comprising between 6 and 8 weeks after childbirth^3^. The evolutionary pressure for survival forces parents to provide care and well-being to their offspring^4^, leading to new demands on the familial context and major changes in social relationships^2^. These changes can modulate specific social cognition domains. Social cognition refers to “the set of mental operations underlying social interactions”^5^, which includes processes implicated in perceiving, interpreting, and generating responses to the intentions and behaviors of others^5,6^. Among these, empathy plays a key role throughout puerperium, as it proves crucial to acknowledge and address the infant’s needs in the absence of verbal communication^7^. Surprisingly, however, social cognition abilities in parents of newborns (PNs) have not been previously studied.

Neurobiological and endocrine adaptations during pregnancy and postpartum include an increase in hormones (e.g., prolactin, oxytocin (OXY), progesterone) and the expression of specific hormonal neuroreceptors^8,9^, which prepare the body for childbirth, nursing, and upbringing^10^. Indeed, some authors propose that pregnancy and postpartum involve cognitive reorganizations^10^ and an increase in maternal sensitivity^7,8^. These changes affect how women respond to emotional information in their environment and how they shape their behaviors in response to exclusive stimuli of motherhood or mother-baby interactions^8^. In fact, increased emotional reactivity reflects the sensitivity of the mother to external stimuli and has a direct effect on the way the mother responds to her surroundings, in particular, to the signals from her baby^10^.

In line with these findings, several studies have found changes in cortical volume associated to sexual steroid hormones that regulate neuronal morphology^7,11^. Indeed, after birth and two years afterwards, women exhibit reduced gray matter volumes in regions subserving social cognition (i.e., amygdala, insula, precuneus, superior temporal and medial prefrontal areas)^11^. In addition, greater neural activity in these regions has been observed in response to the visual signals of the babies. These brain changes predict measures of postpartum maternal attachment, suggesting an adaptive process serving the transition into motherhood^11^. Moreover, functional neuroimaging studies have shown that hypo-oxytocinergic non-breastfeeding mothers as well as non-parents exhibit decreased responses to visual signals of the babies in areas that have OXY receptors or direct connections to oxytocin-sensitive areas^7^.

Even though maternal caregiving involves neurobiological processes related to pregnancy and labor, the father’s brain also adapts to the parental role as well^12,13^. In particular, testosterone and prolactin levels of fathers are key mediators of paternal behavior: lower testosterone and higher prolactin levels have been associated with higher sympathy and paternal alertness as well as positive responses to infant cries^14^. Immediately after birth, and during the first few months, parents focus on the infants’ physical and psychological needs, increasing their primary maternal preoccupation, which will lead to developing emotional bonds while fostering empathy and emotional recognition^15^. Research has shown an association of maternal and paternal OXY levels with parent-infant synchrony^16^, showing increased levels during the first six months after birth^13,17^. OXY levels are related with the parent-specific repertoire^12^; they have a crucial role in the regulation of pro-social and affiliative behavior (e.g., mating, pair-bond formation, maternal/parental behavior, attachment), facilitating social cognition in humans^18,19^.

Moreover, PNs experience widespread changes in varied behavioral domains, such as contextual threat detection, emotional expression, and social attachment^1,20,21^. In fact, increased maternal sensitivity to mother-baby interactions during postpartum^8,15^ is associated with amplified protective mechanisms for warning and defense^1^ and hypervigilance towards signals of threat and potential harm^8^. These changes influence how PNs process emotional information in the environment and how they shape their behaviors accordingly^8,10^.

Baby stimuli activate the “parenting” brain circuits, which share cortico-limbic circuits that regulate other forms of social attachment and behavior, and they are more active during early post-partum^7^. Also, parental sensitivity is critically related to modulations of executive functions, including attentional control, working memory, and flexible task-switching^15^, emotion regulation, reward/motivation system, and parental thoughts^15^. Parents experience a dynamic change in their thoughts and behavior and this intense and chronic mental focus activates motivational-reward pathways and empathy^15^.

Taken together, these findings suggest that PNs might experience major adaptations in their social cognition abilities. Although no experimental study has examined the issue, these changes might prove particularly conspicuous in particular affective domains, such as empathy and social emotions^22^. Essential for human interaction^23,24^, these two domains underpin the welfare and interactive dynamics of social groups, and they may be modulated by circumstances beyond self-experiences and interests^23–25^, such as the transition to parenthood. Empathy comprises the “capacity to share and understand the subjective experience of others in reference to oneself”^23^. This complex construct involves at least three primary components: (a) an affective response to another person (i.e., sharing the other person’s emotional state); (b) a cognitive capacity to take the perspective of other individual; and (c) regulatory mechanisms that monitor the origins of self- and other-feelings^26,27^. Specifically, empathy may be heightened in PNs as it favors caregiving and the perception of social cues from infants^15,22^. Furthermore, higher empathy levels in parents increase secure attachment in children^22^.

Also, substantial changes might be expected in the realm of social emotions. Unlike non-social emotions, social emotions depend on other people’s feelings, actions or thoughts^28–30^. Besides, non-social emotions are often provoked by stimuli with a direct physiological relevance, while social emotions appear in social interactions^31^. Envy and *Schadenfreude*–pleasure at others’ misfortunes– are two social emotions of which experience may be modulated by the biobehavioral changes characterizing transition to parenthood.

Envy refers to the discomfort associated with another person’s good fortune, while *Schadenfreude* denotes the perceiver’s pleasure at another’s unfortunate situation^32,33^. These two social emotions are fundamental in regulating social behavior and maintaining stability of social interactions^34,35^. Furthermore, both envy and *Schadenfreude* are boosted when individuals make upward comparisons^35^. Also, these emotions are involved in stabilizing tensions associated with experiencing inferior roles in hierarchical social contexts^32,34,36^. Moreover, both emotions are related. Specifically, *Schadenfreude* is more likely to appear when a misfortune happens to an envied person^33,37^. Besides, these emotions are manifested in the affective appraisal of situations that foreground other people’s fortune relative to social norms, notions of justice, and social welfare^38^. In puerperal stages, PNs increase their capacity to signal and understand social and emotional cues that could affect the baby’s well-being^22^ –an effect that might, in turn, modulate the experience of social emotions.

To trigger envy or *Schadenfreude*, previous studies e.g.^33,34,38–40^, have used experimental tasks in which fortunate or unfortunate events happen to fictional individuals. In these tasks, after reading the scenarios, participants report (by means of scales) how much envy or pleasure (*Schadenfreude*) they felt for the characters. Besides, facial electromyography (EMG) has been used to detect micro-movements related to the expression of envy and *Schadenfreude*), given its relevance to overcome social desirability biases proper to subjective social emotion instruments^41–43^. *Schadenfreude* expressions have been related with increased activity in the *zygomaticus major*^41,42^ and the *orbicularis oculi*^41^, alongside decreased activity in the *corrugator supercilii*^41^. In particular, envied targets elicit greater responses on the *zygomaticus major* when paired with negative (relative to positive) events^42^. Still, no previous studies have compared EMG correlates of these two social emotions (envy and *Schadenfreude*) nor assessed their modulations in PNs.

Against these antecedents, we evaluated empathy and social emotions in mothers and fathers of newborns (immediately postpartum, only a few hours after giving birth), compared to a control group. In addition, we assessed whether empathy and social emotion outcomes in PNs were influenced by executive functions (EFs), given that they have been associated both domains in other populations^38,44^. Also, considering that social cognition may be modulated by other experiential factors, we controlled for perceived stress levels, depression symptoms, and hours of sleep. In brief, this study aims to add new information for the emergent agenda regarding socio-emotional adaptations during pregnancy and puerperium.

## Methods

### Participants

The study comprised 115 healthy adults from the same geographical area, namely: 55 PNs (31 women, 24 men) and 60 controls (35 women, 25 men). We excluded four PNs and four control participants because they not complete the totality of the protocol. Mothers or biological fathers of newborns were recruited from Hospital Universitario San Ignacio (Bogota, Colombia). For each newborn, only one parent (the mother or the father) participated in this study. They were evaluated after labor, within the first 6 and 24 hours after childbirth and once breastfeeding had been initiated. No participants had a history of neurological or psychiatric disorders, or of alcohol or drug abuse. Controls neither had children, a romantic relationship nor have had sexual intercourse in the previous 48 hours. Also, all control women were in the luteal phase of the menstrual cycle or using hormonal contraceptives. Participants in both groups were matched for sex, age, and depression symptoms (Table 1). However, PNs and controls differed in educational level, executive functioning, perceived stress levels, and hours of sleep during the previous week (Table 1). These differences were included as covariates in all social cognition analyses. All subjects participated voluntarily and signed informed consent prior to the evaluation in agreement with the Helsinki declaration. The study was approved by the Ethics Committees of Universidad de Los Andes and Hospital Universitario San Ignacio.

**Table 1:** Demographic data, EFs and other relevant variables.

	PNs (*n* = 55)	Controls (*n* = 60)	PNs vs. controls	95% CI	
	Mean/SD	Mean/SD	*p-*value	Lower	Upper
**Demographics**					
Age (years)	28.40/5.16	26.97/6.38	0.202	−0.779	3.646
Sex (F:M)	31:24	35:25	0.831		
Education (years)	14.16/3.99	16.22/2.60	0.001	−3.287	−0.819
**Executive functions assessment**					
Total IFS score	23.42/3.06	26.0/1.87	<0.001	−3.509	−1.655
**Relevant variables**					
Hours of Sleep	5.31/1.86	6.03/1.29	0.014	−1.286	−0.149
ZDS	35.13/8.48	35.82/7.48	0.644	−3.64	2.260
PSS	26.28/7.80	22.45/8.34	0.013	0.822	6.834

PNs: parents of newborns; IFS: INECO Frontal Screening battery; ZDS: Zung Depression Scale; PSS: Perceived Stress Scale.

### Instruments

The control group was evaluated in a laboratory room. The PNs were evaluated in a separate quiet room at the Gynecology Service of the Hospital Universitario San Ignacio.

### Empathy

Empathy was assessed with the Interpersonal Reactivity Index (IRI)^45^. This self-report questionnaire measures four dimensions of affective and cognitive empathy, namely: (a) fantasy, i.e., the capacity or tendency to identify with characters of novels and films; (b) perspective taking, i.e., the ability to understand the position and arguments of others; (c) empathic concern, i.e., feelings of sympathy, compassion, and concern (e.g., the capacity to feel what others feel); and (d) personal distress, i.e., feelings of anxiety or discomfort when observing negative experiences to third parties. The IRI comprises 28 situations, seven for each subscale, which are individually rated on a scale from 0 (does not describe me well) to 4 (describes me very well). Four scores were derived from this scale, one for each dimension.

### Social emotions

We employed a previously reported^38–40^ computerized task involving sentences designed to trigger envy (e.g., “She/he managed to get accepted at the university because she/he is the son/daughter of the Dean”) and *Schadenfreude* (e.g., “She/he was discovered as being corrupt and he/she was denounced”) by depicting everyday justice-related situations. The task was divided into two blocks: the first one comprised sentences that evoked envy, and the second one contained sentences that evoked *Schadenfreude* –this sequencing was adopted given that envy and *Schadenfreude* are interdependent and the former could promote the latter^33^. Furthermore, five neutral events were included in each block for control purposes. In each block, participants read 20 different sentences presented in pseudorandomized order on a computer screen. After reading each sentence, participants indicated having read the full sentence by pressing the space bar. Then, they reported, with the keyboard, the intensity of their displeasure (envy) or pleasure (*Schadenfreude*) using a 9-point Likert scale ranging from 1 (low emotional intensity) to 9 (high emotional intensity). Thus, the inter-stimulus interval was ~8 seconds (*M* = 7.90, *SD* = 2.40).

Envy and *Schadenfreude* stimuli had similar linguistic properties in terms of length of the sentences, complexity, and grammatical structure (see details in S2 and Supplementary Table 1).

### Electromyography recordings

Following reported procedures^41^, we used EMG to detect micro-movements of facial expressions related to envy and *Schadenfreude* in a subsample of participants (45 PNs and 23 controls). Registers of remaining participants were excluded because they had completed less than 80% of the trials due to technical problems and/or the presence of excessive signal noise. We used a Micromed SD LTM EXPRESS 64 device. Before electrode placement, the skin was cleansed with alcohol and rubbed with electrode paste. Four bipolar electrodes were used to measure electrical activity while participants performed the experimental task. As in previous EMG studies on social emotions^41,42^, the electrodes were located on the left side of the participants’ face, over four muscles: (a) *corrugator supercili*, (b) *depressor supercilii*, (c) *orbicularis oculi*, and (d) *zygomaticus major*. Additionally, one electrode was placed on the forehead as a ground signal. The *corrugator supercilii* and *depressor supercilii* enable crinkling of the eyebrow, the *orbicularis oculi* closes the eyelids and allows the person to blink, and the *zygomaticus major* helps in smiling by pulling the perioral muscles upward^46^.

Electrodes were placed in accordance with the Guidelines for Human Electromyographic Research^47^. The ground electrode was placed at the midline approximately 3–4 cm superior to the upper borders of the inner brows. For the *corrugator supercilii*, one electrode was affixed above the brow and the other electrode was positioned 1 cm lateral to, and slightly superior to, the first one on the border of the eyebrow. The *depressor supercilii* shares a muscle with the *corrugator supercili* and thus and electrode, the first one mentioned above. We used a jumper in order to use a single electrode for this same muscle. The second electrode for the *depressor supercilii* was placed at a 1-cm distance on the side of the nasal bone. The first electrode in the *orbicularis oculi* was affixed 1 cm inferior to the commissure of the eye fissure, and the other one was located 1 cm medial to, and slightly inferior to the first, so that the electrode pair is parallel to the lower edge of the eyelid. Finally, to measure the electric activity of the *zygomaticus major*, we placed one electrode midway along an imaginary line joining the cheilion and the preauricular depression, and the second electrode was placed 1 cm inferior and medial to the first one along the same imaginary line^47^. To hold the electrodes, we used Ten20 Conductive Paste and hypoallergenic tape. The signals were converted to digital data at a sampling rate of 256 Hz^48^.

Several pilot tests were done to verify there was no electrical interference that could affect the EMG signal. Participants were asked to move as little as possible so as not to affect the EMG signal through body movements.

### Data analysis

#### Behavioral data

Demographic, cognitive state, and behavioral data were compared among groups via independent sample *t*-tests. Chi-square tests were applied to analyze categorical variables (i.e., sex). All reported results were adjusted with Benjamini-Hochberg correction for multiple comparisons^49^. Given that biobehavioral changes associated to parenthood differ between women and men^8,9^, and considering that mode of delivery may affect hormonal levels (e.g., OXY) associated with maternal behavior^50^, dependent variables yielding significant between-group differences were subjected to additional subgroup analyses comparing: (a) mothers vs. fathers, (b) women with vaginal delivery vs. C-section, and (c) women with and without induced labor. Alpha levels were set at 0.05 for all analyses. Effect sizes were calculated through Cohen’s d, with cut-offs of 0.20, 0.50, and 0.80 for small, middle, and large effects, respectively^51^.

A post hoc sensitivity analysis was performed using G*power^52^. Results of this analysis showed that, assuming a power of 0.80 and α level of 0.05, our sample size was sufficient to detect a medium effect size (*d* = 0.52, critical *t* = 1.98).

Given that educational level, executive functioning, perceived stress levels and hours of sleep may be confounding variables, we included these measures in analyses of covariance (ANCOVA) to control for their influence on empathy and social emotion outcomes. Effect sizes for these analyses were calculated through partial eta squared (η^2^).

Also, we conducted multiple regression analyses to explore whether empathy and social emotion outcomes abilities were associated with educational level, EFs, sex, perceived stress levels or mean hours of sleep during the previous week. Specifically, we estimated regression models in which measures yielding significant between-group differences were considered as dependent variables. Group, sex, IFS scores, hours of sleep, and perceived stress levels were included as predictive factors in all models. The latter two measures were included as predictors, given that we found differences between groups in perceived stress levels and hours of sleep during the previous week (see details in S3).

#### EMG data

Following previous procedures^53^, we filtered raw data using a high-pass filter of 20 Hz to eliminate low-frequency noise. Similarly, a notch-filter of 60 Hz was implemented to attenuate electromagnetic noise^47^. After the filtering process, the response to each stimulus was represented as the average signal of a two-second post-trigger time interval (2 seconds after the stimuli presentation). As in previous EMG studies^54^, all signals were standardized considering baseline activity levels elicited through neutral stimuli. The neutral response activity from each time bin was subtracted from the corresponding activity levels triggered in envy and *Schadenfreude* situations. We did not consider traditional pre-trigger muscle activity because, in our case, it corresponded to the rating of the previous sentence and it could add undesired facial expressions.

After baseline correction, and in line with previous procedures^55–57^, EMG data were transformed into z-scores to remove variability and allow for direct comparisons between conditions. For each participant, z-scores were calculated based on the participants’ averages and the mean and standard deviation of the whole sample. If the z-score of a participant’s muscle activity was deviant (i.e., more than 2 *SD* away from the sample’s mean), the associated EMG data was excluded. Whole data from six PNs and three control participants were excluded using this data cleaning. After this procedure, the final sample size for EMG data was 39 PNs and 20 controls.

Then, we conducted 2 (condition: envy and *Schadenfreude*) × 2 (group: PNs and controls) ANOVAs to identify muscular regions that reacted differently to envy compared with *Schadenfreude* and their differences between groups. We conducted an independent analysis for each muscular region. Tukey’s HSD post-hoc tests were used (when appropriate) to examine differences within each condition. Effect sizes were calculated through partial eta squared (η^2^).

## Results

### Empathy

**Figure 1:**
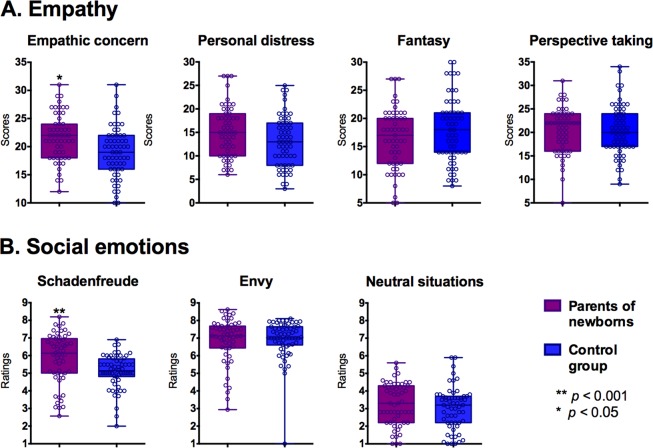
(**A**) Empathy levels in both groups, as tapped through the IRI subscales. (**B**) Social emotion ratings in both groups, for envy *and Schadenfreude*.

PNs showed higher scores than controls in empathic concern (*t* = 3.26, *p* = 0.004, *d* = 0.61). This difference remained significant after adjusting for educational level (*F* (1,112) = 9.99, *p* = 0.002, η^2^ = 0.082), executive functioning (*F* (1,112) = 7.35, *p* = 0.008, η^2^ = 0.061), stress levels (*F* (1,111) = 11.818, *p* < 0.001, η^2^ = 0.094) and hours of sleep (*F* (1,109) = 12.846, *p* < 0.001, η^2^ = 0.104). Also, a marginal between-group difference emerged in the personal distress subscale (*t* = 2.08, *p* = 0.08, *d* = 0.38). This effect was significant after controlling for hours of sleep (*F* (1,109) = 4.888, *p* = 0.029, η^2^ = 0.043). However, the difference disappeared after controlling for executive functioning (*F* (1,112) = 2.21, *p* = 0.140, η^2^ = 0.019), educational level (F (1,12) = 2.61, p = 0.109, η^2^ = 0.022) and stress levels (*F* (1,111) = 2.539, *p* = 0.114, η^2^ = 0.022) Fantasy (*t* = −1.62, *p* = 0.13, *d* = −0.10) and perspective taking (*t* = −0.01, *p* = 0.98, *d* = 0.24) subscales yielded non-significant differences between groups. See Fig. 1A and Supplementary Table 2.

Affective empathy levels did not differ significantly between mothers and fathers or as a function of gynecological and obstetric variables measured (see details in S4 and S5 and Supplementary Tables 3, 4 and 5).

### Social emotions

#### Behavioral results

Compared to controls, PNs exhibited higher *Schadenfreude* ratings (*t* = 3.125, *p* = 0.003, *d* = 0.60), a pattern that remained significant after adjusting for educational level (*F* (1,112) = 11.92, *p* < 0.001, η^2^ = 0.095), executive functioning (*F* (1,112) = 5.85, *p* = 0.017, η^2^ = 0.049), stress levels (*F* (1,111) = 8.229, *p* = 0.005, η^2^ = 0.068), and hours of sleep *F* (1,109) = 8.982, *p* = 0.003, η^2^ = 0.076). Nevertheless, no significant between-group differences were observed in ratings of envy (*t* = −0.28, *p* = 0.77, *d* = −0.05) or neutral situations (*t* = 0.98, *p* = 0.45, *d* = 0.18) –see Fig. 1B and Supplementary Table 2. Analysis of envy scores revealed one outlier participant (2 *SD*s below the group’s mean), but results remained highly similar upon removal of this subject (see details in S6).

Finally, *Schadenfreude* ratings did not differ significantly between mothers and fathers or as a function of gynecological and obstetric variables measured (see details in S4 and S5 and Supplementary Tables 3, 4 and 5).

To establish how specific our results were to PNs, and how independent they are to the testing context, we performed a complementary analysis comparing PNs vs. a second control group (*n* = 34) evaluated in the same experimental context as the experimental group. Results showed that, relative to both control groups, PNs exhibited higher EC, PD, and *Schadenfreude* levels. The results of these complementary analyses have been reported in “Supplementary Data” (S7).

#### EMG results

A significant effect of emotion showed higher activity in the *depressor* region for envy relative to *Schadenfreude* (*F* (1,57) = 10.77, *p* = 0.001, η^2^ = 0.16). No significant effect of group was observed (*F* (1,57) = 0.21, *p* = 0.64, η^2^ = 0.003). Besides, there was no significant interaction between emotion and group (*F* (1,57) = 1.40, *p* = 0.24, η^2^ = 0.02).

**Figure 2:**
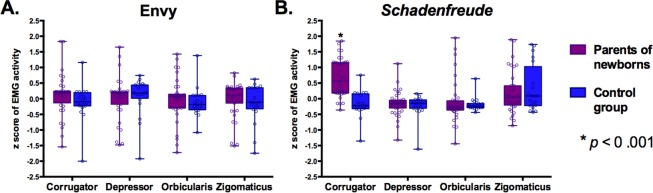
Differences between groups in z-scores of EMG activity for each muscle and emotion. Asterisk indicates significant differences between groups.

A tendency for an effect of emotion was observed for the *corrugator supercilii* region (*F* (1,57) = 3.51, *p* = 0.06, η^2^ = 0.06), showing a higher activity of this region for *Schadenfreude* compared to envy. We found a main effect of group (*F* (1,57) = 20.91, *p* = 0.00002, η^2^ = 0.27) showing that PNs had higher activity on this muscle than controls. We also found an interaction between group and emotion (*F* (1,57) = 5.14, *p* = 0.02, η^2^ = 0.08). A post-hoc analysis (Tukey HSD, *MS* = 0.35, *df* = 110.22) revealed that, for *Schadenfreude*, PNs showed higher activity in the *corrugator supercilii* region than controls (*p* = 0.0001) (see Fig. 2B). No group difference was found for envy (*p* = 0.99).

For the *zygomaticus major* we found a significant effect of emotion (*F* (1,57) = 10.64, *p* = 0.001, η^2^ = 0.15) showing that activity in this region was higher for *Schadenfreude* than envy. There were no significant effect of group (*F* (1,57) = 0.12, *p* = 0.72, η^2^ = 0.002) or interaction between emotion and group (*F* (1,57) = 2.24, *p* = 0.13, η^2^ = 0.03).

The *orbicularis oculi* region did not show any significant modulations for emotion (*F* (1,57) = 0.14, *p* = 0.70, η^2^ = 0.002), group (*F*(1,57) = 0.60, *p* = 0.43, η^2^ = 0.01) or their interaction (*F* (1,57) = 0.03, *p* = 0.85, η^2^ = 0.0006).

### Association between social cognition outcomes and relevant experiential variables

We estimated four multiple regression models considering *Schadenfreude*, envy and the two affective empathic subscales as dependent variables. We included group, sex, educational level, IFS scores, hours of sleep, and perceived stress levels as predictive factors in the four models. A first model including *Schadenfreude* as dependent variable (*F* (6, 104) = 2.512, *p* = 0.026, R^2^ = 0.076) revealed that group (*p* = 0.029) was the only significant predictor. The second model, including envy scores as dependent variable was not significant (*F* (6, 104) = 1.521, *p* = 0.179, R^2^ = 0.028) (see Supplementary Table 6).

The two subsequent models included empathic concern (*F*(6, 104) = 2.680, *p* = 0.019, R^2^ = 0.084) and personal distress (*F*(6, 104) = 1.683, *p* = 0.132, R^2^ = 0.036) as dependent variables. The only significant predictor was group for emphatic concern with *p* = 0.002 (see Supplementary Table 7).

### Correlations between empathy and social emotions

No association was found between empathy and *Schadenfreude*, including all participants and each group separately. Neither a correlation was found between empathy and envy, considering all participants, PNs, and control groups (see Supplementary Tables 8 and 9).

## Discussion

This is the first study investigating social cognition abilities in PNs. We found that, compared to controls, PNs exhibited higher levels of affective empathy and *Schadenfreude*, the latter pattern being accompanied by increased EMG modulations of the *corrugator supercilii*. These results further our understanding of social cognition changes during the puerperal period.

As expected, PNs showed higher scores than controls in both affective empathy subscales (i.e., empathic concern and personal distress), even after adjusting for executive functioning, educational levels, perceived stress levels and hours of sleep. Conversely, non-significant differences were observed between groups in cognitive empathy. Our results are consistent with previous suggestions^7^ that empathy is a key aspect of parenting, especially because babies’ needs are expressed non-verbally. Specifically, empathic concern and personal distress levels are highly related with the social cognition abilities required to recognize and care for others people’s feelings, and even turn to their aid^45^. In line with our findings, in the first stage of bonding, affective empathy is more important and essential than cognitive empathy^58^. Higher affective empathy levels are involved in better emotional communication, social attachment, and motivation to cooperate^58^. Increased parental empathy^7^ facilitates emotional communication, social attachment, parental caring^58^, and motivation to protect and care for the newborn^1^. Notably, given the nature of our empathy measure, our results suggest that higher affective empathy levels observed in PNs are not limited to parent-baby interactions, but are also present in scenarios involving other individuals.

Regarding social emotions, our results showed increased *Schadenfreude* levels in PNs, which were not explained by executive functioning, educational levels, stress levels or hours of sleep. By contrast, envy levels were similar between groups. This pattern may be associated with the multiple hormonal, emotional, and biological changes that take place during pregnancy and puerperium. However, as endocrine, physiological or other biological measures were not included in this study, interpretations about the relevance of these factors should be cautions. A possible explanation for the selective differences in *Schadenfreude* observed in PNs might be the pleasurable nature of this emotion^33,59^ and its strong relationship to reward mechanisms^40^, indexed by increased engagement of the ventral striatum^33^. In fact, this brain region, along with others (e.g., thalamus, hippocampus and amygdala), is crucially involved in oxytocinergic dynamics^33,60^. Previous studies suggest that the neurohor mone OXY may partly account for variations in parent-infant interactions^7^. Higher OXY levels may be associated a wide range of emotions and social behaviors, such as raising children, trusting others, attacking potential outsiders and competing with rivals, which can lead to trust and generosity, but at the same time to increased *Schadenfreude*^61^. Null differences in envy might be explained by the fact that, unlike *Schadenfreude*, this is a non-gratifying emotion that implies feelings of dissatisfaction with another person’s good fortune^40^. In fact, envy implies greater neuronal activity in pain circuits rather than in the reward and pleasure systems^62^. Promisingly, this new hypothesis, derived from our behavioral results, paves the way for new cross-methodological studies. Future studies should include neuroimaging measures as well as OXY and other hormones levels in order to test this interpretation.

Additionally, our social emotion task comprised a group of justice-related scenarios. Accordingly, the higher *Schadenfreude* scores in PNs could reflect an enhanced sensitivity to track unfair situations and respond to scenarios in which those situations are punished. In fact, *Schadenfreude* might play a positive role when unfair social situations are sanctioned^63^, which aligns with a widespread human trend to punish unfair or social inappropriate situations –namely, altruistic punishment^64^, a behavior that is likely underpinned by negative emotions towards defectors. Note, in this sense, that higher OXY levels seem to increase altruistic punishment behavior, by rendering cooperation and promoting cohesion in social groups^65^. Arguably, PNs exhibited higher *Schadenfreude* for unfair or threatening scenarios as an expression of an increased sensitivity to track social threats. Conversely, although the envy situations described unfair and inappropriate social situations, the lack of differences between PNs and controls might reflect the role of control mechanisms in the former, favoring proactive punishment over mere unpleasantness in the face of unfair social scenarios.

These interpretations are further supported by our EMG results. In line with previous EMG studies^41,42^, we found that activity of the *zygomaticus major* activity was higher for *Schadenfreude* than envy responses. Consistent with previous research^41^, this finding suggests that participants seem to exhibit a subtle contortions similar to those involved in the act of smiling when a misfortune happens to another person. In addition, we found that in control participants the *depressor* muscle activity was higher for envy than *Schadenfreude*. *Depressor supercilii* activity show increased activity in response to negative facial stimuli (i.e., angry faces)^66^. Increased activity of this muscle may be explained by the fact the envy stimuli employed here involve situations related to negative feelings of deservingness (e.g., a young man got a better test score for being the son of a professor) or morality/legality (e.g., a politician takes a vacation using taxpayers’ money). Furthermore, EMG results revealed that implicit muscular correlates of *Schadenfreude* involve higher activity in the *corrugator supercilii* for PNs than controls. Note that modulation of the *corrugator supercilii* indexes the disapproval of an action^54^, a process noticeably involved in *Schadenfreude* responses. Considering that linguistic properties of stimuli may affect the *zygomaticus major* and *corrugator supercilii* activities^67^, sentences for envy and *Schadenfreude* conditions were controlled in terms of length, complexity, and grammatical structure. Thus, our behavioral and EMG results can hardly be attributed to differences in the linguistic properties of both conditions stimuli.

Taken together, our results suggest that affective empathy and emotional reactivity to unfair or threating social situations (*Schadenfreude*) are increased in PNs. Accordingly, social cognition changes seem present in mothers and fathers of newborns, irrespective of type of delivery. In general, PNs seem more sensitive to the influence of others and to salient social cues, which are crucial for parental bonding. These patterns align with previous studies showing that the neural circuits underlying emotions in response to socially valued scenarios are partly targeted by the oxytocinergic system^65^. In fact, exogenous OXY levels correlate positively with levels of empathy^68,69^ and *Schadenfreude*^61^. Note, in this sense, that elevated OXY levels in PNs^13,17^ may selectively facilitate social cognition in certain conditions^68,69^ and increase the salience of social cues^61^. Consistent with previous suggestions^70,71^, it has been proposed that OXY has a dual effect on parental behavior, insofar as it inhibits aggression towards the offspring while promoting territoriality as well as aggressive and defensive behaviors against outsiders. As biological measures were not included in the present study, future research should correlate serum or salivary levels of OXY and other hormones levels (e.g., prolactin, OXY, progesterone, estrogen, and cortisol) of pregnant/puerperium women and their partners with performance in social cognition tasks. Furthermore, given that the relatively small sample size for EMG data is a limitation of this study, further studies should investigative social cognition domains, their associated muscle responses, and their peripheral and neural correlates in larger samples of PNs.

We have found a particular pattern of results as we observed at the same time increased affective empathy and *Schadenfreude* levels in PNs. Although it has been theoretically suggested that *Schadenfreude* is a counter-empathic emotion^35,72^, there is no direct evidence supporting such an association. Indeed, our results showed that empathy and *Schadenfreude* are not correlated. Thus, our results suggest that empathy and social emotions changes observed in PNs seem to be dissociable.

Besides, in our study we assessed the role of negative mood factors and cognitive factors in modulating *Schadenfreude* and empathy effects in PNs. In particular, we conducted covariation analyses to assess the extent in which depression and stress modulate the experience of *Schadenfreude*. These covariation analyses did not reach significant effects suggesting that increased *Schadenfreude* in PNs is not directly explained by the mediation of other emotional or cognitive changes occurred at afterbirth stages. In addition, puerperium is considered as a particular intense emotional milestone in PNs’ life, usually associated with emotional changes and stress^73,74^. However, this milestone could be also accompanied by happy mood and the experience of positive emotions such as joy, contempt or happiness. A potential limitation in our study was that we did not measure the role of positive emotions and happy mood in the experience of *Schadenfreude*. To date, the state-of-art of studies assessing *Schadenfreude* has shown dissociable neurocognitive and behavioral mechanisms underlying *Schadenfreude* and positive emotions^33,34,63,75^. Furthermore, note that this is arguably one of the reasons why previous studies on *Schadenfreude* have not controlled for the effects of joy or happy mood^33,36,42,43,59,76^. However, previous studies have revealed the complexity of positive emotions and its influences on secondary emotions^77^. Those influences could also impact on the experience of social emotions, including *Schadenfreude*. New studies should assess the extent to which dispositional emotions or instant and evoked emotional states could affect the intensity and experience of social emotions and social cognition in particular biological states as puerperium or pregnancy. Besides, the group of effects on social emotions and empathy observed in PNs could also be affected by general changes on emotional reactions including fear, anger, and happiness among other emotional manifestations. Future studies also should control the effects of primary emotions on the social emotions and empathy. Finally, the difference in the experimental testing contexts between PNs and controls represents a limitation of our study. However, the results of the complementary analyses (with a control group evaluated in the same setting as the experimental groups) suggest that our pattern of results is not explained by differences in testing sites. Future studies should use specific designs to evaluate the potential impact of different contextual variables on performance.

In sum, this report offers unprecedented evidence that PNs exhibit increased emotional reactivity, characterized by an exacerbation of affective empathy and *Schadenfreude*. These results open a new agenda to examine changes in social cognition and their relationship with neuroendocrine phenomena.

## Supplementary information

Supplementary information.

## Data Availability

The data that support the findings of this study are available from the corresponding author upon reasonable request.

## References

[1] Anderson MV, Rutherford MD (2012). Cognitive reorganization during pregnancy and the postpartum period: An evolutionary perspective. Evol Psychol..

[2] Mosek-Eilon V, Feldman R, Hirschberger G, Kanat-Maymon Y (2013). Infant reminders alter sympathetic reactivity and reduce couple hostility at the transition to parenthood. Dev Psychol..

[3] Diniz CP, Araujo Junior E, Lima MM, Guazelli CA, Moron AF (2014). Ultrasound and Doppler assessment of uterus during puerperium after normal delivery. J Matern Fetal Neonatal Med..

[4] Swain JE (2012). Parenting and Beyond: Common Neurocircuits Underlying Parental and Altruistic Caregiving. Parent Sci Pract..

[5] Penn DL, Sanna LJ, Roberts DL (2008). Social cognition in schizophrenia: an overview. Schizophr Bull..

[6] Adolphs R (1999). Social cognition and the human brain. Trends Cogn Sci..

[7] Swain JE, Lorberbaum JP, Kose S, Strathearn L (2007). Brain basis of early parent-infant interactions: Psychology, physiology, and *in vivo* functional neuroimaging studies. J Child Psychol Psychiatry..

[8] Pearson RM, Lightman SL, Evans J (2009). Emotional sensitivity for motherhood: Late pregnancy is associated with enhanced accuracy to encode emotional faces. Horm Behav..

[9] Russell JA, Douglas AJ, Ingram C (2001). D.Brain preparations for maternity – adaptive changes in behavioral and neuroendocrine systems during pregnancy and lactation. Prog Brain Res..

[10] Gollan JK, Rosebrock L, Hoxha D, Wisner KL (2014). Changes in attentional processing and affective reactivity in pregnancy and postpartum. Neurosci Neuroecon..

[11] Hoekzema E (2017). Pregnancy leads to long-lasting changes in human brain structure. Nat Neurosci..

[12] Abraham E (2014). Father’s brain is sensitive to childcare experiences. PNAS..

[13] Gordon I, Zagoory-Sharon O, Leckman JF, Feldman R (2010). Oxytocin and the Development of Parenting in Humans. Biol Psychiatry..

[14] Fleming AS, Corter C, Stallings J, Steiner M (2002). Testosterone and prolactin are associated with emotional responses to infant cries in new fathers. Horm Behav..

[15] Swain JE (2014). Approaching the biology of human parental attachment: Brain imaging, oxytocin and coordinated assessments of mothers and fathers. Brain Res..

[16] Feldman R (2012). Sensitive Parenting Is Associated with Plasma Oxytocin and Polymorphisms in the OXTR and CD38 Genes. Biol Psychiatry..

[17] Feldman R (2007). Parent–Infant Synchrony: Biological Foundations and Developmental Outcomes. Curr Dir Psychol Sci..

[18] Bartz A, Hollander J (2006). E. The neuroscience of affiliation: Forging links between basic and clinical research on neuropeptides and social behavior. Horm Behav..

[19] Ross HE, Young LJ (2009). Oxytocin and the neural mechanisms regulating social cognition and affiliative behavior. Front. Neuroendocrinol..

[20] Feldman R, Weller A, Zagoory-Sharon O, Levine A (2007). Evidence for a Neuroendocrinological Foundation of Human Affiliation: Plasma Oxytocin Levels across Pregnancy and the Postpartum Period Predict Mother-Infant Bonding. Psychol Sci..

[21] Brunton PJ, Russell JA (2008). The expectant brain: adapting for motherhood. Nat. Rev. Neurosci..

[22] Boorman RJ, Creedy DK, Fenwick J, Muurlink O (2019). Empathy in pregnant women and new mothers: a systematic literature review. J Reprod Infant Psyc..

[23] Decety J (2011). The neuroevolution of empathy. Ann N Y Acad Sci..

[24] Haidt J (2008). Morality. Perspect Psychol Sci..

[25] Baez, S. *et al*. Men, women…who cares? A population based study on sex differences and gender roles in empathy and moral cognition. *PLOS ONE*. **12**, 10.1371/journal.pone.0179336 (2017). 10.1371/journal.pone.0179336 PMC5478130 28632770

[26] Decety J, Jackson PL (2004). The functional architecture of human empathy. Behav Cogn Neurosci Rev..

[27] Decety J, Michalska KJ, Kinzler KD (2012). The contribution of emotion and cognition to moral sensitivity: a neurodevelopmental study. Cereb. Cortex..

[28] Leary, M. R. Affect, cognition, and the social emotions In *Feeling and thinking* (ed. Forgas, J. P.) 331–356 (Cambridge U. Press, 2000).

[29] Tracy JL, Robins R (2004). Putting the self into self-conscious emotions: A theoretical model. Psychol. Inq..

[30] Burnett S, Blakemore SJ (2009). The development of adolescent social cognition. Ann N Y Acad Sci..

[31] Britton JC (2006). Neural correlates of social and nonsocial emotions: An fMRI study. NeuroImage..

[32] Dvash J, Gilam G, Ben-Ze’ev A, Hendler T, Shamay-Tsoory SG (2010). The envious brain: The neural basis of social comparison. Hum Brain Mapp..

[33] Takahashi H (2009). When your gain is my pain and your pain is my gain: neural correlates of envy and schadenfreude. Science..

[34] Cikara M, Fiske ST (2013). Their pain, our pleasure: stereotype content and schadenfreude. Ann N Y Acad Sci..

[35] Baez, S., García, A. M. & Santamaria-Garcia, H. The missing link in *Neuroscience and social science* (eds. Ibanez, A. Sedeno, L. & García, A. M.) (Springer Nature, 2017).

[36] Jankowski KF, Takahashi H (2014). Cognitive neuroscience of social emotions and implications for psychopathology: examining embarrassment, guilt, envy, and schadenfreude. Psychiatry clin neurosci..

[37] Zaki J (2015). Envy, politics, and age. Emotion..

[38] Santamaría-García H (2017). A lesion model of envy and *Schadenfreude*: legal, deservingness and moral dimensions as revealed by neurodegeneration. Brain..

[39] Baez S (2018). Corticostriatal signatures of schadenfreude: evidence from Huntington’s disease. J Neurol Neurosurg Psychiatry..

[40] Baez S (2016). Your misery is no longer my pleasure: Reduced schadenfreude in Huntington’s disease families. Cortex..

[41] Boecker L, Likowski KU, Pauli P, Weyers P (2015). The face of schadenfreude: Differentiation of joy and schadenfreude by electromyography. Cogn. Emot..

[42] Cikara M, Fiske ST (2012). Stereotypes and schadenfreude: Affective and physiological markers of pleasure at outgroup misfortunes. Soc Psychol Personal Sci..

[43] McNamee M (2003). Schadenfreude in sport: Envy, justice, and self-esteem. J Philos..

[44] Decety, Jackson (2004). The functional arquitecture of human. Behav Cogn Neurosci Rev..

[45] Davis MH (1983). Measuring individual differences in empathy: Evidence for a multidimensional approach. J. Pers. Soc. Psychol..

[46] Westbrook, K. E. & Varacallo, M. Anatomy, Head and Neck, Facial Muscle in *StatPearls* (StatPearls Publishing, 2019). 29630261

[47] Fridlund AJ, Cacioppo JT (1986). Guidelines for Human Electromyographic Research. Psychophysiology..

[48] Hayes KJ (1960). Wave analyses of tissue noise and muscle action potentials. J Appl Physiol..

[49] Benjamini Y, Hochberg Y (1995). Controlling the false discovery rate: A practical and powerful approach to multiple testing. J. R. Statist. Soc. Ser. B Stat. Methodol..

[50] Swain JE (2008). Maternal brain response to own baby-cry is affected by cesarean section delivery. J Child Psychol Psychiat..

[51] Lachenbruch, P. A. *Statistical Power Analysis for the Behavioral Sciences*. (American Statistical Association, 1989).

[52] Faul F, Erdfelder E, Lang AG, Buchner A (2007). G*Power 3: a flexible statistical power analysis program for the social, behavioral, and biomedical sciences. Behav Res Methods..

[53] Fino E, Menegatti M, Avenanti A, Rubini M (2016). Enjoying vs. smiling: Facial muscular activation in response to emotional language. Biol Psychol..

[54] Hart, B., Struiksma, M. E., van Berkum, J. J. A. & van Boxtel, A. Emotion in stories: Facial EMG evidence for both mental simulation and moral evaluation. *Front Psychol*. **9**, 10.3389/fpsyg.2018.00613 (2018). 10.3389/fpsyg.2018.00613 PMC5937160 29760671

[55] Hess U, Blairy S (2001). Facial mimicry and emotional contagion to dynamic emotional facial expressions and their influence on decoding accuracy. Int J Psychophysiol..

[56] Murata, A., Saito, H., Schug, J., Ogawa, K. & Kameda, T. Spontaneous Facial Mimicry Is Enhanced by the Goal of Inferring Emotional States: Evidence for Moderation of “Automatic” Mimicry by Higher Cognitive Processes. *PLoS One*. **11**, 10.1371/journal.pone.0153128 (2016). 10.1371/journal.pone.0153128 PMC4824486 27055206

[57] Neta M, Norris CJ, Whalen PJ (2009). Corrugator muscle responses are associated with individual differences in positivity-negativity bias. Emotion..

[58] Decety N, Berntson C (2012). A neurobehavioral evolutionary perspective on the mechanisms underlying empathy. Prog Neurobiol..

[59] Dvash, J. & Shamay-Tsoory, S. G. Envy and schadenfreude: The neural correlates of competitive emotions in *DNA to social cognition* (2011).

[60] Campbell A (2008). Attachment, aggression and affiliation: The role of oxytocin in female social behavior. Biol Psychol..

[61] Shamay-Tsoory SG (2009). Intranasal Administration of Oxytocin Increases Envy and Schadenfreude (Gloating). Biol Psychiatry..

[62] Lieberman MD (2007). Social Cognitive Neuroscience: A Review of Core Processes. Annu. Rev. Psychol..

[63] Van Dijk WW, Ouwerkerk JW, Goslinga S (2006). When people fall from grace: reconsidering the role of envy in Schadenfreude. Emotion..

[64] Fehr E, Gachter S (2002). Altruistic punishment in humans. Nature..

[65] Aydogan G (2017). Oxytocin promotes altruistic punishment. Soc Cogn Affect Neurosci..

[66] Lundqvist LO (1995). Facial EMG reactions to facial expressions: a case of facial emotional contagion?. Scand J Psychol..

[67] Topolinski S, Likowski K, Weyers P, Strack F (2009). The face of fluency: Semantic coherence automatically elicits a specific pattern of facial muscle reactions. Cogn. Emot..

[68] Bartz JA (2010). Oxytocin Selectively Improves Empathic Accuracy. Psychol. Sci..

[69] Shamay-Tsoory SG, Abu-Akel A (2016). The Social Salience Hypothesis of Oxytocin. Biol Psychiatry..

[70] Dębiec J (2005). Peptides of love and fear- Vasopressin and oxytocin modulate the integration of information in the amygdala. Bioessays..

[71] Pedersen CA (2004). Biological Aspects of Social Bonding and the Roots of Human Violence. Ann N Y Acad Sci..

[72] Cikara, M., Bruneau, E. G. & Saxe, R. R. Us and Them: Intergroup Failures of Empathy. *Curr. Dir. Psychol. Sci*. **20**, 149–153, 10.1177_0956797610397667 (2011).

[73] Kendell RE, McGuire RJ, Connor Y, Cox JL (1981). Mood changes in the first three weeks after childbirth. J Affect Disord.

[74] Turton P (2006). Psychological impact of stillbirth on fathers in the subsequent pregnancy and puerperium. Br J Psychiatry.

[75] van Dijk, W. W. & Ouwerkerke, J. W. *Schadenfreude. Understanding Pleasure at the Misfortune of* Others (ed. van Dijk, W. W & Ouwerkerke, J. W) (Cambridge University Press, 2017).

[76] Smith RH, Powell CAJ, Combs DJY, Schurtz DR (2009). Exploring the when and why of Schadenfreude. Soc Personal Psychol Compass..

[77] Fredrickson BL (1998). What Good Are Positive Emotions?. Rev Gen Psychol.

