# Supplementary material for: The unique social sense of puerperium: Increased empathy and Schadenfreude in parents of newborns
Source: Sci Rep. 2020 Apr 1;10:5760. doi: 10.1038/s41598-020-62622-7 (PMC7113282; doi:10.1038/s41598-020-62622-7)
Supplement: Supplementary file 1 — Supplementary information. [file 41598_2020_62622_MOESM1_ESM.pdf]

The unique social sense of puerperium: Increased empathy and *Schadenfreude* in parents of newborns

Ana-María Gómez-Carvajal [1,2,+,] Hernando Santamaría-García [3, 4,+] Adolfo M. García [5,6,7,8] Mario Valderrama [9], Jhony Mejia [9], Jose Santamaría-García [1], Mateo Bernal [1], Jaime Silva [10], Agustín Ibáñez [5, 6, 10, 11,12] and Sandra Baez [1, +,*]

[1] Universidad de los Andes, Bogotá, Colombia
[2] School of Medicine and Health Sciences, Neuroscience Research Group NEUROS, Universidad del Rosario, Bogotá, Colombia.
[3] Memory and cognition Center, Intellectus. Hospital Universitario San Ignacio, Bogotá, Colombia
[4] Pontificia Universidad Javeriana, Departments of Physiology, Psychiatry and Aging Institute, Bogotá, Colombia
[5] Universidad de San Andrés, Buenos Aires, Argentina.
[6] National Scientific and Technical Research Council (CONICET), Buenos Aires, Argentina
[7] Faculty of Education, National University of Cuyo (UNCuyo), Mendoza, Argentina
[8] Departamento de Lingüística y Literatura, Facultad de Humanidades, Universidad de Santiago de Chile, Santiago, Chile
[9] Department of Biomedical Engineering, Universidad de los Andes, Bogotá, Colombia
[10] Department of gynecology and obstetrics, Hospital Universitario San Ignacio, Bogotá, Colombia
[10] Universidad Autónoma del Caribe, Barranquilla, Colombia
[11] Center for Social and Cognitive Neuroscience (CSCN), School of Psychology, Universidad Adolfo Ibáñez, Santiago de Chile, Chile
[12] Australian Research Council Center of Excellence in Cognition and its Disorders, Sydney, Australia.
+ Equal contribution
* Corresponding author

Corresponding author

Sandra Baez, MS, PhD
Universidad de los Andes
sj.baez@uniandes.edu.co
Phone: +571 3394999 Ext.: 5560
Fax: +57(1) 3324539
Cra. 1 #18a-12, Bogotá, Colombia

Supplementary data

S1. Executive function assessment. The INECO Frontal Screening (IFS) battery is a sensitive tool for detecting executive dysfunction in clinical and healthy populations [1-4]. It includes the following eight subtests: (1) motor programming (Luria series, "fist, edge, palm"); (2) conflicting instructions (hitting the table once when the administrator hits it twice, or hitting it twice when the administrator hits it only once); (3) motor inhibitory control; (4) numerical working memory (backward digit span); (5) verbal working memory (months backwards); (6) spatial working memory (modified Corsi tapping test); (7) abstraction capacity (inferring the meaning of proverbs), and (8) verbal inhibitory control (modified Hayling test). Its maximum possible score is 30 points.

S2. We controlled the linguistic properties of envy and *Schadenfreude* stimuli (see Supplementary Table 1). All stimuli in the envy and *Schadenfreude* blocks consisted of declarative affirmative sentences, with their main verb in active voice and past tense (more precisely, *pretérito perfecto indefinido*). Also, all sentences in both sets comprised two clauses (standing in either paratactic or hypotactic relation) with a strictly systematic syntactic pattern (i.e., [tacit] subject + verb + optional complement).

In addition, both type of stimuli were equal in terms of length (number of words: $t\,(14) = 0$, $p = 1.00$) and total number of content words (verbs, nouns, adjectives, and adverbs: $t\,(14) = 0$, $p = 1.00$). In addition, we used the EsPal database [5] to control for other psycholinguistic properties of the content words (verbs, nouns, adjectives, and adverbs) included in each type of stimuli. Envy and *Schadenfreude* stimuli were also similar in terms of word frequency ($t\,(89) = 0.80$, $p = 0.42$), number of letters ($t\,(89) = -1.07$, $p = 0.28$), number of syllables ($t\,(89) = 1.51$, $p = 0.88$), number of substitution neighbors ($t\,(89) = -0.84$, $p = 0.40$), Levenshtein's distance (level of similarity in terms of number of deletions, insertions, or substitutions required to transform one word into another) ($t\,(89) = 0.59$, $p = 0.55$), familiarity ($t\,(13) = -0.25$, $p = 0.80$), imageability ($t\,(13) = -1.08$, $p = 0.29$), and concreteness ($t\,(13) = -0.61$, $p = 0.54$). Note that for the last three properties, data for all content words included in the stimuli sentences was not available in the EsPal database.

S3. No differences were found between groups in depression levels ($t = -0.463$, $p = 0.644$, $d = -0.086$). However, PNs reported higher stress levels than controls ($t = 2.523$, $p = 0.13$, $d = 0.473$). Also, compared to controls, PNs reported having had fewer hours of sleep during the previous week ($t = -2.500$, $p = 0.014$, $d = -0.474$).

S4. Subgroup comparison between mothers and fathers. No differences between subgroups were found in the affective empathy subscales, namely, empathic concern ($t = -0.640$, $p = 0.525$, $d = -0.174$) and personal distress ($t = -0.160$, $p = 0.873$, $d = -0.044$).

Regarding social emotions, non-significant differences were found between mothers and fathers in *Schadenfreude* levels ($t = -0.325$, $p = 0.747$, $d = -0.088$). See Supplementary Table 2.

S5. Subgroup comparison according with gynecological and obstetric variables. Non-significant differences were found in any affective subscale of the empathy scale in terms of type of delivery [empathic concern (t = 0.557, p = 0.582, d = 0.200); personal distress (t = 1.108, p = 0.277, d = 0.398)] or mode of labor (induced or not induced) [empathic concern (t = 0.679, p = 0.503, d = 0.244); personal distress (t = -1.038, p = 0.308, d = -0.373)].

Non-significant differences were found in *Schadenfreude* as a function of type of delivery (t = -1.595, p = 0.121, d = -0.573) or mode of labor (t = 0.366, p = 0.717, d = 0.131). See Supplementary Tables 3 and 4.

S6. No between-group differences were found in envy scores after removing the outlier subject (t = -0.83, p = 0.40, d = -0.15).

S7. Empathy. PNs showed higher scores than participants in the second control group in empathic concern (t = 3.125, p = 0.003, d = 0.60), a pattern that remained significant after adjusting for educational level (F (1,86) = 13.75, $p < 0.001$, $\eta^2 = 0.01$), executive functioning (F (1,86) = 8.34, p = 0.005, $\eta^2 = 0.08$), stress levels (F (1,86) = 12.90, p = 0.0001, $\eta^2 = 0.13$), and hours of sleep F (1,86) = 14.05, p = 0.0001, $\eta^2 = 0.14$).

A marginal between-group difference emerged in the personal distress subscale (t = 1.86, p = 0.06, d = 0.40). Fantasy (t = 0.04, p = 0.96, d = 0.010) and perspective taking (t = 0.24, p = 0.81, d = 0.05) subscales yielded non-significant differences between groups.

Social emotions. Compared to the second control group, PNs exhibited higher *Schadenfreude* ratings (t = 2.66, p = 0.009, d = 0.58), a pattern that remained significant after adjusting for educational level (F (1,86) = 7.99, $p < 0.006$, $\eta^2 = 0.08$), stress levels (F (1,86) = 5.19, p = 0.02, $\eta^2 = 0.05$), and hours of sleep F (1,86) = 5.71, p = 0.01, $\eta^2 = 0.06$). The difference was marginal after adjusting for executive functioning (F (1,86) = 2.87, p = 0.08, $\eta^2 = 0.03$). Nevertheless, no significant between-group differences were observed in ratings of envy (t = -0.72, p = 0.47, d = -0.15) or neutral situations (t = 0.89, p = 0.37, d = 0.19).

Supplementary Table 1. Linguistic properties of envy and *Schadenfreude* stimuli

Linguistic property	Envy	*Schadenfreude*
	Mean /*SD*	Mean /*SD*
**Length**		
Number of words	10.67 /1.54	10.67 / 2.49
**Complexity**		
Number of verbs	2.20 / 1.64	2.60 / 0.63
Number of nouns	2.67 / 0.72	2.13 / 0.83
Number of adjectives	0.80 / 0.56	0.67 / 0.61
Number of adverbs	0.47 / 0.51	0.73 / 0.59)
Total number of content words	6.13 /1.06	6.13 /1.59
**Linguistic properties of content words**		
Word frequency	212123.9/ 1038395.1	134101.7 / 487350.9
Number of letters	6.19 / 2.32	6.57 / 2.22
Number of syllables	2.68 / 0.98	2.68 /0.91
Number of substitution neighbors	0.36 / 0.75	0.39 / 0.93
Levenshtein's distance	1700000 / 596949.1	1649444.4 / 502549.3
Familiarity	6113.5 /185.4	6176.8 / 112.8
Imageability	4992.0 / 745.1	5402.5 / 1054.9
Concreteness	4661.8 /973.4	4831.5 / 263.9

Supplementary Table 2. Descriptive data for empathy and social emotion outcomes in both groups.

	PNs (n = 55)	Controls (n = 60)	PNs vs. controls	95% CI for Cohen's *d*	
	Mean / *SD*	Mean / *SD*	*p*-value	Lower	Upper
**Empathy**					
FS	16.44 / 5.45	18.20 / 6.11	0.13	-0.671	0.065
PT	20.38 / 4.99	20.4 / 5.4	0.98	-0.369	0.362
EC	21.67 / 4.16	19.03 / 4.47	0.004	0.234	0.983
PD	15.20 / 5.38	13.10 / 5.44	0.08	0.018	0.757
**Social emotions**					
Schadenfreude	5.84 / 1.44	5.11 / 0.94	0.003	0.232	0.981
Envy	6.86 / 1.29	6.93 / 1.09	0.77	-0.420	0.312
Neutral sentences	3.26 / 1.19	3.04 / 1.19	0.45	-0.183	0.550

PNs: parents of newborns; FS: fantasy scale; PT: perspective taking; EC: empathic concern; PD: personal distress. All *p* values were adjusted using Benjamini and Hochberg corrections.

Supplementary Table 3. Descriptive data for empathy and social emotion outcomes in mothers and fathers.

	Mothers (n = 31)	Fathers (n = 24)	Mothers vs. fathers	95% CI for Cohen's *d*	
	Mean / *SD*	Mean / *SD*	*p*-value	Lower	Upper
**Empathy**					
EC	21.36 / 4.03	22.08 / 4.38	0.525	-0.707	0.361
PD	15.097 / 5.36	15.33 / 5.52	0.873	-0.576	0.490
**Social emotions**					
Schadenfreude	5.787 / 1.53	5.915 / 1.36	0.747	-0.621	0.445

EC: empathic concern; PD: personal distress.

Supplementary Table 4. Descriptive data for empathy and social emotion outcomes considering type of delivery

	C-section (n = 15)	Vaginal (n = 16)	C-section vs. vaginal	95% CI for Cohen's *d*	
	Mean / *SD*	Mean / *SD*	*p*-value	Lower	Upper
**Empathy**					
EC	20.93 / 3.06	21.75 / 4.84	0.582	-0.508	0.905
PD	14.0 / 4.766	16.13 / 5.818	0.277	-0.317	1.107
**Social emotions**					
Schadenfreude	6.227 / 1.078	5.374 / 1.788	0.121	-1.288	0.151

EC: empathic concern; PD: personal distress

Supplementary Table 5. Descriptive data for empathy and social emotion outcomes considering mode of labor (induced vs. not induced).

	Induced labor (n = 15)	Non-induced labor (n = 16)	Induced vs. non-induced labor	95% CI for Cohen's d	
	Mean / *SD*	Mean / *SD*	p-value	Lower	Upper
**Empathy**					
EC	21.867/ 3.603	20.875/ 4.455	0.503	-0.465	0.949
PD	14.067/ 5.625	16.063/ 5.079	0.308	-1.081	0.341
**Social emotions**					
Schadenfreude	5.891 / 1.396	5.688 / 1.678	0.717	-0.575	0.835

EC: empathic concern; PD: personal distress

Supplementary Table 6. Coefficients of the multiple regression models of social emotions

Variables	Model I<br>DV: *Schadenfreude*				Model II<br>DV: Envy			
	β	*P*	95% CI		β	*p*	95% CI	
			Lower	Upper			Lower	Upper
Group	-0.241	0.029	-1.155	-0.064	0.005	0.964	-0.486	0.508
Sex	0.104	0.276	-0.215	0.747	-0.234	0.018	-0.970	-0.093
Education levels	0.141	0.160	-0.021	0.128	0.199	0.248	-0.028	0.108
IFS total score	-0.128	0.235	-0.151	0.038	-0.004	0.972	-0.087	0.084
Hours of sleep	0.034	0.722	-0.130	0.187	-0.090	0.352	-0.213	0.076
Perceived stress levels	0.130	0.186	-0.010	0.050	-0.051	0.613	-0.034	0.020

DV: dependent variable; IFS= INECO frontal screening

Supplementary Table 7. Coefficients of the multiple regression models of affective empathy

Variables	Model III DV: empathic concern				Model IV DV: personal distress			
	β	*p*	95% CI		β	*p*	95% CI	
			Lower	Upper			Lower	Upper
Group	-0.345	0.002	-5.058	-1.175	-0.127	0.254	-3.832	1.023
Sex	-0.061	0.519	-2.272	1.153	-0.093	0.338	-3.180	1.103
Education levels	0.028	0.777	-0.227	0.303	-0.111	0.278	-0.514	0.149
IFS total score	-0.052	0.624	-0.419	0.253	-0.032	0.772	-0.481	0.358
Hours of sleep	0.156	0.099	-0.091	1.039	0.062	0.520	-0.477	0.936
Perceived stress levels	-0.141	0.138	-0.185	0.026	0.156	0.123	-0.028	0.235

DV: dependent variable; IFS= INECO frontal screening

Supplementary Table 8. Correlation between *Schadenfreude* and empathy

	All participants		PN's group		Control group	
	Pearson's r	*p*	Pearson's r	*p*	Pearson's r	*p*
Schadenfreude-FS	0.013	0.91	0.137	0.91	-0.029	0.82
Schadenfreude-PT	-0.062	0.91	0.018	0.91	-0.175	0.68
Schadenfreude-EC	-0.011	0.91	-0.097	0.91	-0.124	0.68
Schadenfreude-PD	0.031	0.91	-0.015	0.91	- 0.044	0.82

FS: fantasy; PT: perspective taking; EC: empathic concern; PD: personal distress. All *p* values were adjusted using Benjamini and Hochberg corrections.

Supplementary Table 9. Correlation between envy and empathy

	All participants		PN's group		Control group	
	Pearson's r	*p*	Pearson's r	*p*	Pearson's r	*p*
Envy-FS	-0.051	0.50	0.027	0.84	-0.136	0.44
Envy-PT	-0.231	0.08	-0.302	0.08	-0.164	0.44
Envy-EC	-0.073	0.50	-0.037	0.84	-0.101	0.44
Envy-PD	-0.099	0.50	-0.069	0.84	-0.125	0.44

FS: fantasy; PT: perspective taking; EC: empathic concern; PD: personal distress. All *p* values were adjusted using Benjamini and Hochberg corrections.

Supplementary references

1 Baez, S. *et al.* The utility of IFS (INECO Frontal Screening) for the detection of executive dysfunction in adults with bipolar disorder and ADHD. *Psychiatry Res.* **216**, 269-276, doi:10.1016/j.psychres.2014.01.020 (2014).

2 Bocanegra, Y. *et al.* Syntax, action verbs, action semantics, and object semantics in Parkinson's disease: Dissociability, progression, and executive influences. *Cortex* **69**, 237-254, doi:10.1016/j.cortex.2015.05.022 (2015).

3 Torralva, T., Roca, M., Gleichgerrcht, E., Lopez, P. & Manes, F. INECO Frontal Screening (IFS): a brief, sensitive, and specific tool to assess executive functions in dementia. *J Int Neuropsychol Soc.* **15**, 777-786, doi:10.1017/S1355617709990415 (2009).

4 Gonzalez-Gadea, M. L. *et al.* Emotion recognition and cognitive empathy deficits in adolescent offenders revealed by context-sensitive tasks. *Front. Hum. Neurosci.* **8**, 850, doi:10.3389/fnhum.2014.00850 (2014).

5 Duchon, A., Perea, M., Sebastian-Galles, N., Marti, A. & Carreiras, M. EsPal: one-stop shopping for Spanish word properties. *Behav. Res. Methods.* **45**, 1246-1258, doi:10.3758/s13428-013-0326-1 (2013).
